# The impact of clinicopathologic and surgical factors on relapse and pregnancy in young patients (≤40 years old) with borderline ovarian tumors

**DOI:** 10.1186/s12885-018-4932-2

**Published:** 2018-11-21

**Authors:** Chenyan Fang, Lingqin Zhao, Xi Chen, Aijun Yu, Liang Xia, Ping Zhang

**Affiliations:** 1 Department of Gynecological Oncology, Zhejiang Cancer Hospital, 1 Banshan East Road, Hangzhou, 310022 Zhejiang Province China; 2 Department of Neurosurgery, Zhejiang Cancer Hospital, 1 Banshan East Road, Hangzhou, 310022 Zhejiang Province China

**Keywords:** Borderline ovarian tumors, Fertility sparing surgery, Disease-free survival, Pregnancy

## Abstract

**Background:**

Fertility sparing surgery has been extensively performed among patients with borderline ovarian tumors due to their age and favorable prognosis. Nevertheless, the prognosis and obstetric outcomes in these patients remain uncertain. Thus, the current study was carried out to evaluate the oncological safety and fertility benefits of different fertility sparing surgery subtypes and various clinicopathological parameters.

**Methods:**

Young borderline ovarian tumor patients with an age of ≤40 years, who were admitted and treated in Zhejiang Cancer Hospital from January 1996 to December 2016, were enrolled in this study and reviewed retrospectively. The prognostic and obstetric effects of clinicopathological and surgical variables were evaluated using univariate/multivariate analyses and survival curves.

**Results:**

A total of 92 eligible patients were enrolled in the analysis. Among these patients, 22 (24%) patients showed recurrence after a median follow-up of 46.5 months. Within the fertility sparing surgery group, patients at advanced stage (≥stage II), of serous type, with micropapillary and bilateral tumors were associated with a higher recurrence rate and a shorter recurrence interval. In terms of different modalities of fertility sparing surgery, adnexectomy was remarkably favored over cystectomy-including (*P* = 0.012); unilateral salpingo-oophorectomy had better prognosis than cystectomy and bilateral cystectomy was favored over unilateral salpingo-oophorectomy+contralateral cystectomy. Univariate Cox regression analysis indicated that the International Federation of Gynecology and Obstetrics stage (≥Stage II), the presence of bilateral and micropapillary lesions, and the application of cystectomy-including surgery were correlated with poorer disease-free survival, while the mucinous type of borderline ovarian tumors was related to improved disease-free survival. In this study, a total of 22 patients attempted to conceive and 15 (68%) of these patients achieved successful pregnancy.

**Conclusions:**

Unilateral salpingo-oophorectomy and bilateral cystectomy should be recommended as the preferred choice of treatment for young patients with unilateral and bilateral borderline ovarian tumor who desire to preserve fertility. In addition, borderline ovarian tumor patients at advanced stage (≥stage II), of serous type, with micropapillary and bilateral tumors should pay more attention to the risk of recurrence. Therefore, these patients should choose fertility sparing surgery carefully and attempt to achieve pregnancy as soon as possible.

## Background

Borderline ovarian tumors were first described by Taylor in 1929 as a “semi-malignant” ovarian tumors [1]. Borderline ovarian tumors are non-invasive neoplasms characterized by atypical epithelial proliferation, nuclear atypia, and a level of mitotic activity between that of benign and invasive cancer. However, destructive stromal invasion has not been seen in borderline ovarian tumors, which are hence referred to as “borderline tumors” or “atypical proliferative tumors”, whereas the term of “tumor of low malignant potential” is no longer recommended in the 2014 World Health Organization (WHO) Classification of Tumors of Female Genital Organs [2]. Borderline ovarian tumors account for approximately 10% to 20% of all epithelial ovarian cancer, whose annual prevalence is 1.8–4.8/100,000 women [3, 4]. Notably, the proportion of borderline ovarian tumors in ovarian malignancies shows an increasing trend [5]. Consistent with that for invasive ovarian cancer, complete staging is the standard criterion for the selection of surgical treatments, including cytology of peritoneal washing, hysterectomy, bilateral salpingo-oophorectomy, omentectomy, and complete resection of peritoneal macroscopic lesions. Nevertheless, borderline ovarian tumors frequently occur in young women and the mean age is 38 years. Most of borderline ovarian tumors patients are diagnosed at the early stage, although even borderline ovarian tumors diagnosed at the advanced stage still have a favorable prognosis [6]. Fertility sparing is an important consideration in planning the treatment for young borderline ovarian tumor patients due to the childbearing age and favorable prognosis [7]. Up to now, fertility sparing surgery has been widely accepted while their safety and feasibility have been demonstrated in numerous studies [8, 9]. However, few studies have compared the prognosis and specific obstetric outcomes among different fertility sparing surgery modalities and clinicopathological factors. Thus, the current study was carried out to investigate the obstetric and oncological outcomes of four fertility sparing surgery modalities in young borderline ovarian tumor patients at a childbearing age. The four fertility sparing surgery modalities included cystectomy versus unilateral salpingo-oophorectomy for unilateral borderline ovarian tumors, and bilateral cystectomy versus unilateral salpingo-oophorectomy + contralateral cystectomy for bilateral borderline ovarian tumors. In addition, the influence of various clinicopathological factors on the recurrence and fertility outcomes of borderline ovarian tumor patients was also examined. The results of this study may assist young borderline ovarian tumor patients in their selection of an optimal treatment.

## Methods

Young women aged ≤40 years that were histologically diagnosed as any type of primary borderline ovarian tumors in Zhejiang Cancer Hospital from January 1996 to December 2016 were retrospectively evaluated in this study. A total of 54 patients undergoing fertility sparing surgery were enrolled in this study, and those with concurrent ovarian cancer or other malignant ovarian tumors were excluded. This study was approved by the Medical Ethics Committee of our hospital. No written informed consent was obtained from all patients due to the retrospective nature of the study. Data were retrospectively retrieved from hospital records and patient charts, including age, tumor size, lesion lateral, International Federation of Gynecology and Obstetrics (FIGO) stage, type of operation, histological subtype, the history of chemotherapy and follow-up information. Meanwhile, obstetric and oncological outcomes were collected by medical record review, telephone interview or out-patient interview.

Patients were staged according to surgical findings and the FIGO criteria (2014). In addition, the histological types of the tumor were determined in accordance with the WHO system (2003). Histopathological information was obtained from pathological specimens, which were evaluated by pathologists experienced in gynecologic pathology. Patients were divided into 4 histological types, including serous, mucinous, endometrioid and mixed types. Micropapillary lesions were diagnosed in a serous borderline tumor containing complex micropapillary structures which demonstrate a filigree pattern. Microinvasion was defined as stromal invasion restricted to an area of no more than 10 mm^2^.

Surgical approaches were selected based on the age of patients, extent of tumor, the demand for pregnancy, the time of diagnosis (intraoperative versus postoperative diagnosis), and the opinion of experienced gynecologic oncologists. Two types of surgical operations were selected in this study, i.e., fertility sparing surgery, which was performed to conserve uterus and at least a portion of one ovary, and radical resections, which included total hysterectomy, bilateral salpingo-oophorectomy, resection with or without the removal of lymph nodes, resection of the greater omentum below the transverse colon, multiple abdominal biopsies, and peritoneal lavage of exfoliated cells. Four fertility sparing surgery modalities were selected in this study, including cystectomy, unilateral salpingo-oophorectomy, bilateral cystectomy and unilateral salpingo-oophorectomy + contralateral cystectomy. According to clinical records, most patients underwent cystectomy first, followed by restaging surgery in our hospital upon the diagnosis of borderline ovarian tumors.

Patients were followed up once every 3 months and 6 months for the first 2 years and year 3–5 after the surgery, respectively, and once a year thereafter. Gynecological examination, abdominal ultrasonography and tumor marker evaluation were recommended in each follow-up. Disease-free survival (DFS, defined as the duration from primary surgery to the first recurrence or the last visit) and overall survival (OS, defined as the duration from primary surgery to death or the last visit) were employed to assess the oncological outcomes.

### Statistical analysis

DFS, recurrence rate and pregnancy rate were selected as the primary outcomes in our study. All statistical analyses were performed using the Statistical Package for Social Sciences (SPSS) statistical software (version 17.0). Categorical data were assessed using chi-square or Fisher’s exact test. Correlations of clinicopathological factors and surgical variables with DFS and obstetric outcomes were assessed using univariate and multivariate Cox regression models, and were expressed as hazard ratios (HR). Meanwhile, recurrence-free interval and survival curves were assessed using the Kaplan-Meier method, whereas statistically significant difference was examined by log rank test. *p* < 0.05 indicated significant difference.

## Results

### Patient characteristics

A total of 92 young borderline ovarian tumor patients aged ≤40 years were analyzed in this study, and their surgical information and clinicopathological characteristics are listed in Table 1.

**Table 1:** Patients’ characteristics

	Total	FSS (N%)	Radical surgery (N%)
Total	92	54 (58.7)	38 (41.3)
FIGO stage			
IA	35 (38)	31 (57.4)	4 (10.5)
IB	9 (9.8)	3 (5.6)	6 (15.8)
IC	18 (19.6)	8 (14.8)	10 (26.3)
II	8 (8.7)	1 (1.9)	7 (18.4))
III	22 (23.9)	11 (20.4)	11 (28.9)
Diameter			
< 10 cm	50 (54.3)	27 (50.0)	23 (60.5)
≥ 10 cm	42 (45.7)	27 (50.0)	15 (39.5)
Histology			
Serous	46 (50)	26 (48.1)	20 (52.6)
Mucinous	38 (41.3)	25 (46.3)	13 (34.2)
Endometrioid	7 (7.6)	2 (3.7)	5 (13.2)
Serous and mucinous	1 (1.1)	1 (1.9)	0 (0)
Lesion lateral			
Left	44 (47.8)	26 (48.1)	18 (47.4)
Right	29 (31.5)	17 (31.5)	12 (31.6)
Bilateral	19 (20.7)	11 (20.4)	8 (21.1)
Rupture			
Yes	17 (18.5)	10 (18.5)	7 (18.4)
No	75 (81.5)	44 (81.5)	31 (81.6)
Micropapillary			
Yes	16 (17.4)	11 (20.4)	5 (13.2)
No	76 (82.6)	43 (79.6)	33 (86.8)
Microinvasion			
Yes	7 (7.6)	5 (9.3)	2 (5.3)
No	85 (92.4)	49 (90.7)	36 (94.7)
Invasive implants			
Yes	4 (4.3)	2 (3.7)	2 (5.3)
No	88 (95.7)	52 (96.3)	36 (94.7)
Pelvic lymph node			
Positive	10 (10.9)	6 (11.1)	4 (10.5)
Negative	82 (89.1)	48 (88.9)	34 (89.5)
Para-aortic lymph node			
Positive	3 (3.3)	2 (3.7)	1 (2.6)
Negative	89 (96.7)	52 (96.3)	37 (97.4)
Lymphadenectomy			
Yes	55 (59.8)	26 (48.1)	29 (76.3)
No	37 (40.2)	28 (51.9)	9 (23.7)
Surgery approach			
Laparoscopy	7 (7.6)	5 (9.3)	2 (5.3)
Laparotomy	85 (92.4)	49 (90.7)	36 (94.7)
Adjuvant chemotherapy			
Yes	10 (10.9)	4 (7.4)	6 (15.8)
No	82 (89.1)	50 (92.6)	32 (84.2)
Staging surgery			
Yes	87 (94.6)	51 (94.4)	36 (94.7)
No	5 (5.4)	3 (5.6)	2 (5.3)
Appendectomy			
Yes	8 (8.7)	5 (9.3)	3 (7.9)
No	84 (91.3)	49 (90.7)	35 (92.1)
Recurrence			
Yes	22 (23.9)	19 (35.2)	3 (7.9)
No	70 (76.1)	35 (64.8)	35 (92.1)
Death	0	0	0

**Fig. 1:**
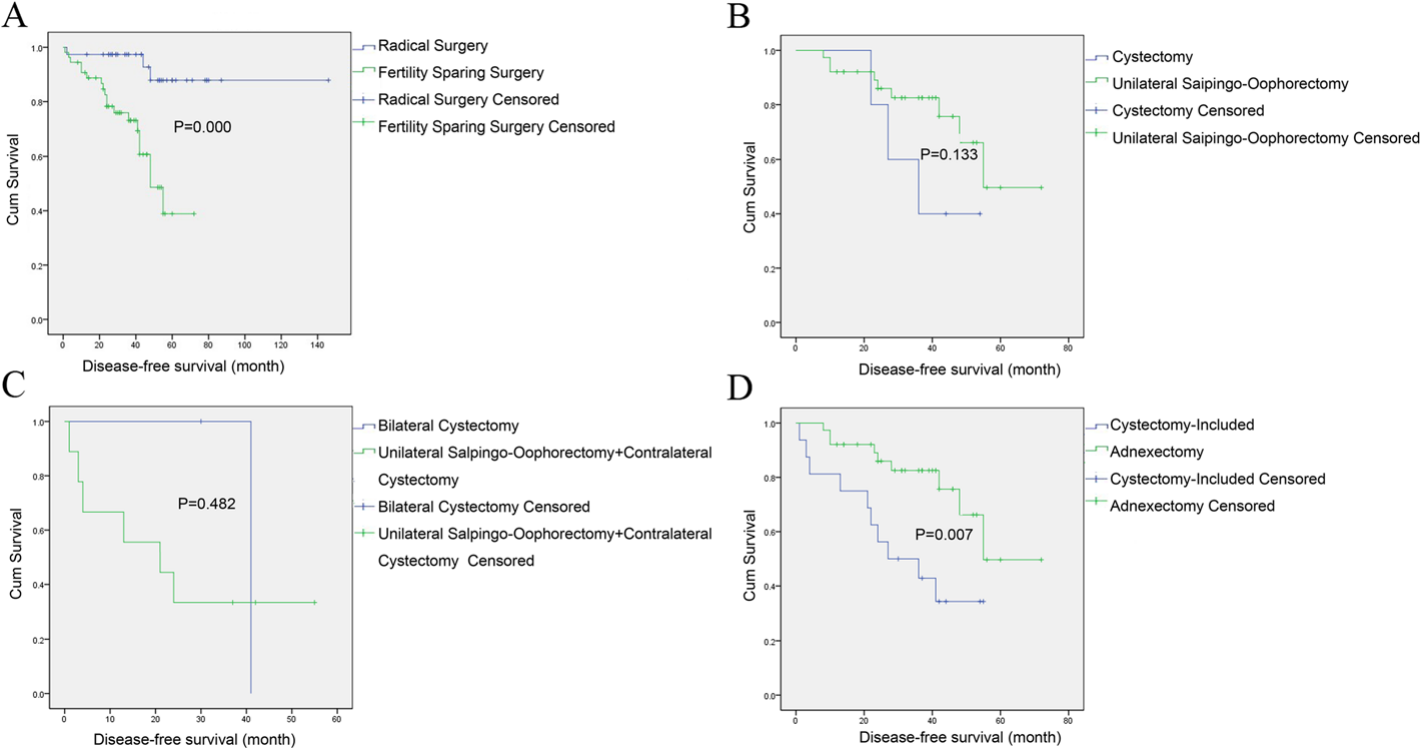
Disease-free survival curves (DFS) of patients undergoing different types of surgeries. **a** Comparison of DFS curves between radical surgery and fertility sparing surgery; **b** Comparison of DFS curves between cystectomy and unilateral salpingo-oophorectomy; **c** Comparison of DFS curves between bilateral cystectomy and unilateral salpingo-oophorectomy + contralateral cystectomy; **d** Comparison of DFS curves between adnexectomy and cystectomy-included

Amongst the enrolled cases, 54 (59%) had undergone fertility sparing surgery while 38 (41%) received radical surgeries. Patients diagnosed at an early stage were more likely to retain their fertility function, especially for those at stage IA (57% vs 11%). In addition, the proportion of patients undergoing lymphadenectomy and postoperative adjuvant chemotherapy were higher in patients undergoing radical surgery (76% versus 48% and 16% versus 7.4%). Patients were followed up for a median of 46.5 months (range, 13–146 months). With regard to the oncological outcomes at the last follow-up, the recurrence rate in patients undergoing fertility sparing surgery was higher than that in those receiving radical surgeries (35% versus 7.9%, *P* = 0.003). In addition, the Kaplan-Meier analysis of DFS is illustrated in Fig. 1a. No death was reported in either group. In addition, the difference of OS between the two groups was not significant (*P* = 0.488).

The majority patients in the fertility sparing surgery group were of FIGO stage I (78%, including 57% of stage IA), while a few cases were of stage II (1.9%) and the remaining cases were of stage III/IV (20%). Besides, serous borderline ovarian tumors (*n* = 26, 48%) were the most common pathological type, followed by mucinous (*n* = 25, 46%), endometrioid (*n* = 2, 3.7%) and serous/mucinous (*n* = 1, 1.9%) borderline ovarian tumors. Among these patients, 11 (20%) had micropapillary lesions, 5 (9.3%) had microinvasion lesions, and 2 (3.7%) had invasive implants in uterovesical peritoneal reflection and sacral ligament. Notably, unilateral borderline ovarian tumors were more common (*n* = 45, 79%, including 48% in the left ovary and 32% in the right ovary) than bilateral borderline ovarian tumors (*n* = 11,20%). 10 patients (19%) developed ovary ruptures, including one intraoperative rupture and nine spontaneous ruptures. Among 26 cases (48%) undergoing lymphadenectomy, 6 (11%) had pelvic lymph node metastases, and 2 (3.7%) had para-aortic lymph node metastases. Among these patients, only 5 (9.3%) underwent laparoscopy and 49 (91%) received laparotomy. Most of the patients (*n* = 51, 94%) underwent staging surgeries while only 4 (7.4%) patients underwent postoperative adjuvant chemotherapies because they were at an advanced FIGO stage, had invasive implants, or had lymph node metastases. In addition, the recurrence rate was high among these patients (19/54, 35%), although no case showed progression into invasive ovarian cancer.

### Recurrence and pregnancy outcomes of borderline ovarian tumors patients in the fertility sparing surgery group

Significant factors for recurrence and pregnancy were assessed using univariate analysis. A variety of clinicopathological and surgical parameters were tested and shown in Table 2.

**Table 2:** The recurrence and pregnancy outcomes of borderline ovarian tumor patients

		Recurrence outcome				Pregnancy outcome	
		N recurrence/Total (%)	*P* value	Recurrence interval (m, mean)	*P* value	N patients achieving/attempting pregnancy (%)	*P* value
FIGO stage	Stage I	11/42 (26.2)	0.016	55	0.036	11/16 (68.8)	1.000
	≥Stage II	8/12 (66.7)		42		4/6 (66.7)	
Diameter	< 10 cm	11/27 (40.7)	0.569	48	0.108	5/11 (45.5)	0.063
	≥10 cm	8/27 (29.6)		N/A		10/11 (90.9)	
Histology	Serous	15/26 (57.7)	0.003	42	0.007	9/13 (69.2)	0.313
	Mucinous	3/25 (12.0)		N/A		6/8 (75.0)	
	Others^a^	1/3 (33.3)		55		0/1 (0)	
Lesion lateral	Unilateral^b^	12/43 (27.9)	0.038	55	0.011	13/18 (72.2)	0.565
	Bilateral	7/11 (63.6)		24		2/4 (50.0)	
Rupture	Yes	3/10 (30.0)	1.000	42	0.743	2/4 (50.0)	0.565
	No	16/44 (36.4)		55		13/18 (72.2)	
Micropapillary	Yes	8/11 (72.7)	0.010	28	0.003	3/4 (75.0)	1.000
	No	11/43 (25.6)		36		12/18 (66.7)	
Microinvasion	Yes	3/5 (60.0)	0.332	48	0.607	1/1 (100.0)	1.000
	No	16/49 (32.7)		55		14/21 (66.7)	
Invasive implants	Yes	2/0 (100.0)	0.119	4	0.116	0/0 (0)	N/A
	No	17/52 (32.7)		48		15/22 (68.2)	
Pelvic lymph node	Positive	2/6 (33.3)	1.000	N/A	0.984	0/3 (0)	0.023
	Negative	17/48 (35.4)		48		15/19 (78.9)	
Lymphadenectomy	Yes	7/26 (26.9)	0.264	55	0.313	6/12 (50.0)	0.074
	No	12/28 (42.9)		48		9/10 (90.0)	
Surgery approach	Laparoscopy	1/5 (20.0)	0.646	36	0.907	2/3 (66.7)	1.000
	Laparotomy	18/49 (36.7)		48		13/19 (68.4)	
Adjuvant chemotherapy	Yes	2/4 (50.0)	0.607	8	0.056	0/2 (0)	
	No	17/50 (34.0)		55		15/20 (75.0)	0.091
Staging surgery	Yes	19/51 (37.3)	0.189	N/A	0.320	14/20 (70.0)	1.000
	No	0/3 (0)		N/A		1/2 (50.0)	
Appendectomy	Yes	0/5 (0)	0.149	N/A	0.287	1/2 (50.0)	1.000
	No	19/49 (38.8)		N/A		14/20 (70.0)	
Fertility sparing surgery (1)	Cystectomy	3/5 (60.0)	0.054	36	0.031	1/2 (50.0)	0.855
	Bilateral cystectomy	1/2 (50.0)		41		1/1 (100.0)	
	Unilateral salpingo-oophorectomy	9/38 (23.7)		55		11/16 (68.8)	
	Unilateral salpingo-oophorectomy + contralateral cystectomy	6/9 (66.7)		21		2/3 (66.7)	
Fertility sparing surgery (2)							
Unilateral^b^	Cystectomy	3/5 (60.0)	0.123	36	0.133	1/2 (50.0)	1.000
	Unilateral salpingo-oophorectomy	9/38 (23.7)		55		11/16 (68.8)	
Bilateral	Bilateral cystectomy	1/2 (50.0)	1.000	41	0.482	1/1 (100.0)	1.000
	Unilateral salpingo-oophorectomy + contralateral cystectomy	6/9 (66.7)		21		2/3 (66.7)	
Fertility sparing surgery (3)	Cystectomy-included	10/16 (62.5)	0.012	27	0.007	4/6 (66.7)	1.000
	Adnexectomy	9/38 (23.7)		55		11/16 (68.8)	

^a^others included endometroid, serous and mucinous; ^b^unilateral included left and right; FIGO, the International Federation of Gynecology and Obstetrics; N/A, not applicable

**Fig. 2:**
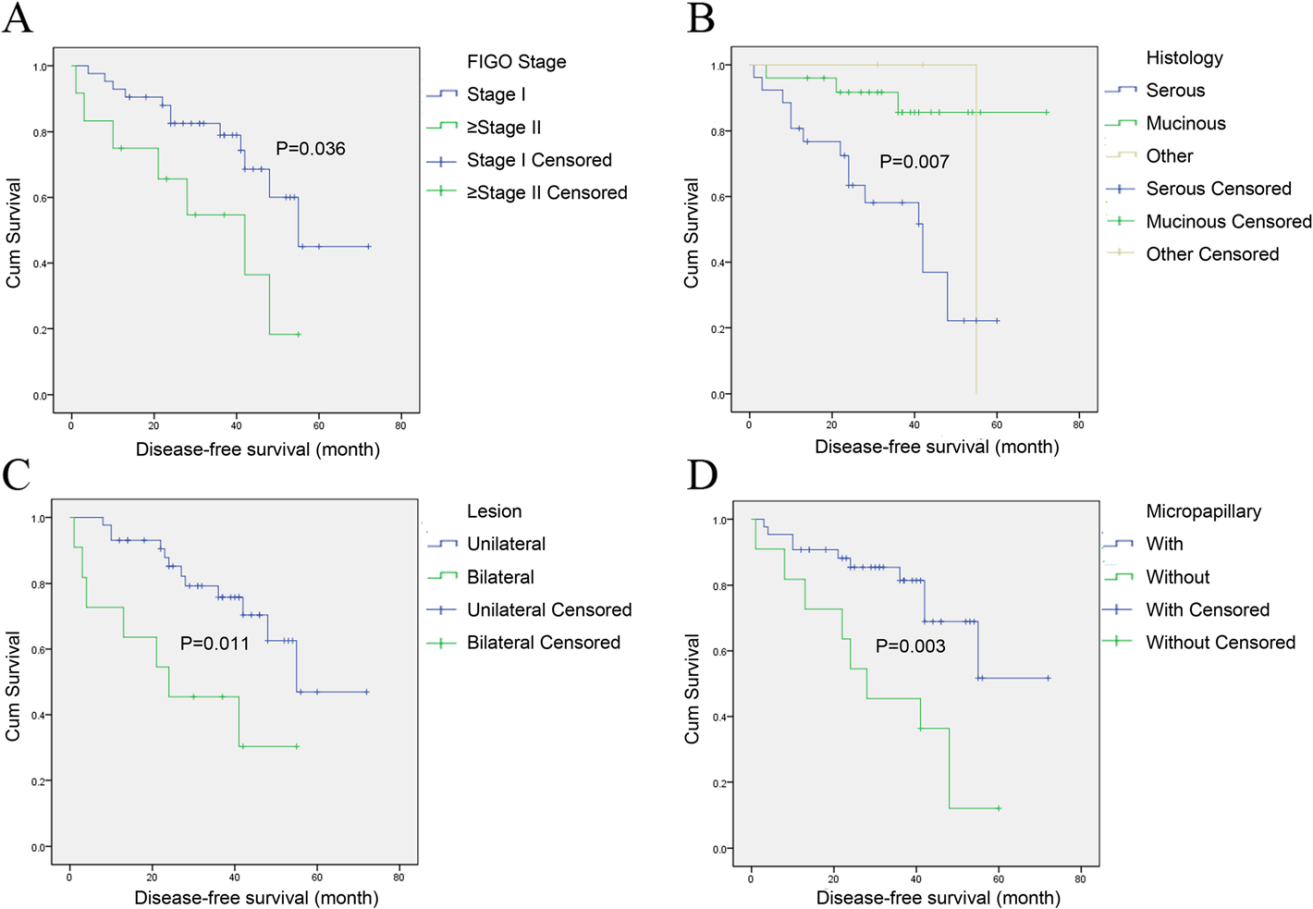
Disease-free survival curves (DFS) for different clinicopathologic factors. **a** Comparison of DFS curves between stage I and ≥ stage II borderline ovarian tumors; **b** Comparison of DFS curves among serous, mucinous and others borderline ovarian tumors; **c** Comparison of DFS curves between unilateral tumor and bilateral tumors; **d** Comparison of DFS curves in borderline ovarian tumors with and without micropapillary lesions

Amongst the clinicopathologic factors, the FIGO stage was a significant factor. Compared with the early stage group (stage I), the recurrence rate was higher (67% versus 26%, *P* = 0.016) and the recurrence interval was shorter (42 months versus 55 months, *P* = 0.036) in the advanced stage group (≥stage II). Meanwhile, the survival curves of patients at the early (stage I) and late (≥stage II) stages are provided in Fig. 2a. As can be seen from the results, the patients with serous borderline ovarian tumors had a higher recurrence rate (*P* = 0.003) and a shorter recurrence interval (*P* = 0.007) (Fig. 2b). The presence of bilateral lesions was another significant factor, as bilateral tumors were associated with a higher recurrence rate (64% versus 28%, *P* = 0.038) and a shorter recurrence interval (24 months versus 55 months, *P* = 0.011) in comparison with patients with unilateral tumors (Fig. 2c). Additionally, the presence of micropapillary lesions was also a significant factor. In particular, the patients with micropapillary lesions were associated with a higher recurrence rate (73% vs 26%, P = 0.01) and a shorter recurrence interval (28 months versus 36 months, *P* = 0.003) (Fig. 2d).

Only one surgical factor was significant, i.e., cystectomy-included versus adnexectomy.

However, none of the above factors markedly affected the pregnancy outcome, since no significant difference in terms of the pregnancy rate was observed among these groups. Most of the patients were associated with a favorable pregnancy rate (> 65%).

### Fertility sparing surgery group

#### Comparison of cystectomy, unilateral salpingo-oophorectomy, bilateral cystectomy and unilateral salpingo-oophorectomy + contralateral cystectomy

In this study, 54 patients underwent fertility sparing surgery, including 5 undergoing cystectomy, 38 receiving unilateral salpingo-oophorectomy, 2 undergoing bilateral cystectomy, and 9 receiving unilateral salpingo-oophorectomy + contralateral cystectomy. The recurrence rates of these patients were 60%, 24%, 50%, and 67% (*P* = 0.054), respectively, and their recurrence intervals were 36, 55, 41 and 21 months (*P* = 0.031), respectively. In addition, the pregnancy rates in these patients were 50%, 69%, 100% and 67% (*P* = 0.855), respectively. Notably, the surgical type of unilateral salpingo-oophorectomy was associated with the lowest recurrence rate and the longest recurrence interval.

#### Cystectomy versus unilateral salpingo-oophorectomy

Compared with the unilateral salpingo-oophorectomy group, the cystectomy group had a higher recurrence rate and a shorter recurrence interval (60% versus 24%, *P* = 0.123/36 months versus 55 months, *P* = 0.133), indicating that the difference between these two groups was not statistically significant. The pregnancy rates in the cystectomy and unilateral salpingo-oophorectomy groups were 50% and 69%, respectively (*P* = 1.000), indicating that the two groups showed no statistically significant difference. The survival curves of these two groups are presented in Fig. 1b.

#### Bilateral cystectomy versus unilateral salpingo-oophorectomy + contralateral cystectomy

The recurrence rate in the unilateral salpingo-oophorectomy + contralateral cystectomy group was 67%, which was higher than 50% in the bilateral cystectomy group (*P* = 1.000). In addition, the recurrence interval in the unilateral salpingo-oophorectomy + contralateral cystectomy group was shorter than that in the bilateral cystectomy group (21 months versus 41 months, *P* = 0.482). The survival curves of these two groups are displayed in Fig. 1c.

Only 2 out of the 11 patients with bilateral borderline ovarian tumors underwent bilateral cystectomy, and only 1 of these two patients attempted pregnancy and was successful, thus resulting in a pregnancy rate of 100% (*N* = 1/1). Meanwhile, the pregnancy rate of patients underwent unilateral salpingo-oophorectomy + contralateral cystectomy was 67% (*N* = 2/3), but their difference in terms of the pregnancy rate was not statistically significant (*P* = 1.000).

#### Cystectomy-including versus Adnexectomy

The surgical types of fertility sparing surgery were divided into two groups, namely, cystectomy-including (cystectomy, bilateral cystectomy, unilateral salpingo-oophorectomy + contralateral cystectomy) and adnexectomy (unilateral salpingo-oophorectomy).

From the results, it can be seen that the surgery approach of adnexectomy showed a significantly better prognosis than the cystectomy-including approach, which was associated with a lower recurrence rate (24% versus 63%, *P* = 0.012) and a longer recurrence interval (55 months versus 27 months, *P* = 0.007). The survival curves of these two groups were examined by Kaplan–Meier analysis and shown in Fig. 1d.

The pregnancy rates in the adnexectomy and the cystectomy-including groups were 69% (*N* = 11/16) and 67% (*N* = 4/6), respectively, although the difference was not statistically significant (*P* = 1.000). Both surgical types displayed a favorable pregnancy rate (> 65%) among borderline ovarian tumor patients.

### Univariate and multivariate cox regression analyses of disease-free survival in the fertility sparing surgery group (Table 3)

Univariate Cox regression analysis suggested that 5 factors were distinctly associated with DFS, including FIGO stage (≥Stage II) (HR = 2.589, 95% credibility interval = 1.017–6.591, *P* = 0.046), histology information (the mucinous type) (HR = 0.184, 95% credibility interval = 0.053–0.636, *P* = 0.007), lesion lateral (bilateral lesions) (HR = 3.135, 95% credibility interval = 1.230–7.989, *P* = 0.017), micropapillary lesions (HR = 3.575, 95% credibility interval = 1.446–8.838, *P* = 0.006), and the type of fertility preserving surgeries (cystectomy-including surgeries) (HR = 3.3, 95% credibility interval = 1.338–8.140, *P* = 0.01).

**Table 3:** Univariate and multivariate analysis of DFS

		Univariate		*P* value	Multivariate		*P* value
		HR	95% confidence intervals		HR	95% confidence intervals	
FIGO stage	Stage I	1					
	≥Stage II	2.589	1.017–6.591	0.046	1.343	0.400–4.506	0.633
Diameter	< 10 cm	1					
	≥10 cm	0.475	0.186–1.214	0.120			
Histology	Serous	1					
	Mucinous	0.184	0.053–0.636	0.007	0.503	0.078–3.238	0.469
	Others^a^	0.341	0.045–2.609	0.300	1.357	0.113–16.267	0.810
Lesion lateral	Unilateral^b^	1					
	Bilateral	3.135	1.230–7.989	0.017	1.239	0.189–8.101	0.823
Rupture	Yes	1.229	0.353–4.284	0.746			
	No	1					
Micropapillary	Yes	3.575	1.446–8.838	0.006	3.380	0.882–12.956	0.076
	No	1					
Microinvasion	Yes	1.461	0.334–6.381	0.614			
	No	1					
Invasive implants	Yes	4.574	0.567–36.912	0.154			
	No	1					
Pelvic lymph node	Positive	1.015	0.233–4.424	0.985			
	Negative	1					
Lymphadenectomy	Yes	0.624	0.245–1.591	0.323			
	No	1					
Surgery approach	Laparoscopy	0.886	0.115–6.830	0.908			
	Laparotomy	1					
Adjuvant chemotherapy	Yes	3.919	0.860–17.861	0.078			
	No	1					
Staging surgery	Yes	22.557	0.002–280,136.902	0.517			
	No	1					
Appendectomy	Yes	0.043	0.000–318.369	0.490			
	No	1					
Fertility sparing surgery (1)	Cystectomy	0.345	0.071–1.676	0.187			
	Bilateral cystectomy	0.527	0.063–4.399	0.554			
	Unilateral salpingo-oophorectomy	0.207	0.077–0.557	0.002			
	Unilateral salpingo-oophorectomy + contralateral cystectomy	1					
Fertility sparing surgery (2)							
Unilateral^b^	Cystectomy	1.806	0.383–8.515	0.455			
	Unilateral salpingo-oophorectomy	1					
Bilateral	Bilateral cystectomy	0.474	0.056–3.985	0.492			
	Unilateral salpingo-oophorectomy + contralateral cystectomy	1					
Fertility sparing surgery (3)	Cystectomy-included	3.300	1.338–8.140	0.010	3.070	0.577–16.344	0.189
	Adnexectomy	1					

^a^others included endometriod, serous and mucinous; ^b^unilateral included left and right; DFS, disease-free survival; FIGO, the International Federation of Gynecology and Obstetrics

A multivariate Cox regression model was constructed in this study to incorporate the statistically significant factors examined in the univariate analysis. Unfortunately, no factor was found to be significantly correlated with DFS.

## Discussion

Borderline ovarian tumors frequently occur in young women and are associated with favorable prognosis. Previous studies have shown that the 5-year and 10-year survival rates of borderline ovarian tumor patients are 95% and 93%, respectively. Among these patients, 5.0–8.0% suffer from recurrence, with 2.0% progressing into invasive ovarian cancer. In addition, the mortality of borderline ovarian tumors is low [10, 11]. Therefore, a conservative surgery is the preferred choice for patients at a reproductive age who desire to preserve fertility. However, Trillsch F et al. [12] reported that the recurrence rate of borderline ovarian tumors in patients undergoing fertility sparing surgery was 10–20%, which was markedly higher than that in those receiving radical surgeries (5.0%). In this study, the recurrence rate of borderline ovarian tumor patients undergoing fertility sparing surgery was also notably higher than that of the patients receiving radical surgeries (35% versus 7.9%, *P* = 0.003), while the Kaplan-Meier analysis of DFS between these two groups also showed statistically significant difference. Zanetta G et al. [11] reported that 7 out of the 189 borderline ovarian tumor patients undergoing fertility sparing surgery developed invasive recurrence. Fertility conservation usually means to retain the uterus and at least one side of the ovaries. Nevertheless, fertility sparing surgery should always be recommended with caution due to the high recurrence rate of borderline ovarian tumors, poor DFS and invasive recurrence of borderline ovarian tumors. Importantly, the balance between oncological and fertile outcomes should be assessed adequately. This study has retrospectively analyzed the oncological and pregnancy outcomes of young borderline ovarian tumor patients with different clinicopathological factors and different types of surgeries. It is expected that this study can help young borderline ovarian tumor patients to select an optimal treatment.

Up to now, numerous studies have implicated various factors, such as histological type (a serous type), the presence of invasive implants, FIGO stage (an advanced stage), the presence of micropapillary lesions, lesion lateral (bilateral tumors), residual disease, and the presence of stromal microinvasion, were associated with the poor prognosis of borderline ovarian tumor patients.

Among previous studies, Chang C et al. [13] reported that the histology type of borderline ovarian tumors might affect the progression-free survival (PFS) of borderline ovarian tumor patients, since the hazard ratio (HR) of serous/mucinous tumors was 2.656, although the difference was not statistically significant (*P* = 0.084). Karlsen NMS et al. [14] also found a negative correlation between the serous histology (*P* = 0.037) and the risk of tumor relapse. On the contrary, no difference was observed in the survival rate between different histological types (serous versus mucinous, endometrioid and mixed tumor; *P* = 0.15) [15], Chen RF et al. [16] demonstrated a favorable prognosis of serous tumors. The authors found that the patients of serous borderline ovarian tumors who underwent fertility sparing surgery had a longer recurrence interval than those with mucinous tumors (35.9 versus 18.5 months, *P* < 0.001). In the analysis of this study, the patients with serous borderline ovarian tumors had a higher recurrence rate (58% versus 12%, *P* = 0.003) and a shorter recurrence interval (*P* = 0.007) than those with mucinous tumors. In addition, the univariate Cox regression analysis also suggested that the histology type (mucinous) can significantly decrease the risk of recurrence (HR = 0.184, *P* = 0.007) after conservative surgeries.

Many articles have shown that higher FIGO stages are always accompanied by higher recurrence rates. Seong SJ et al. [17] indicated that the 5-year survival for stage I borderline ovarian tumor patients was approximately 95% to 97%, while the 5-year survival for stage II-III borderline ovarian tumor patients was only 65% to 87%. Zanetta G et al. [11] found that, compared with women at stage I (15%), those at a more advanced stage had a higher recurrence rate (40%) after conservative surgeries. Meanwhile, the probabilities of lethal recurrence at the early and advanced stages are 0.5% and 2.0%, respectively [18]. However, the difference between different stages in terms of survival rate was not significantly significant (stage I-II versus stage III; *P* = 0.74) [15]. In this study, higher FIGO stages (≥stage II) significantly reduced the DFS while the risk of relapse was increased (*P* < 0.05).

Unfortunately, the impact of micropapillary pattern on serous borderline ovarian tumor patients remains a source of controversy at present. Chen X et al. [19] analyzed 178 borderline ovarian tumors patients and their univariate Cox regression analysis showed that micropapillary pattern was significantly associated with PFS (HR = 3.88, *P* = 0.0008). Similarly, micropapillary pattern has also been reported as an independent prognostic factor for borderline ovarian tumor patients [20, 21]. However, du Bois A et al. [22] found that the micropapillary growth pattern was not evidently associated with the prognosis of borderline ovarian tumor patients. Moreover, some studies have suggested that micropapillary borderline ovarian tumor is more frequently associated with bilateral lesions, advanced stages at diagnosis, invasive implants, lymph node involvement and decreased survival [17, 23]. Based on the results shown in this study, the patients with micropapillary lesions were closely correlated with negative oncological outcomes (including a higher recurrence rate and a shorter recurrence interval) and decreased DFS after fertility sparing surgery (*P* < 0.05).

In addition, Uzan C et al. [24] identified bilateral tumors as a risk factor of recurrence, since the 5-year recurrence-free survival was 71% and 48% in patients with unilateral and bilateral tumors, respectively (*P* = 0.05). In addition, Karlsen NMS et al. [14] also indicated that bilateral tumors were a notable risk factor of recurrence via univariate analysis. Chen RF et al. [16] analyzed 122 borderline ovarian tumor patients undergoing conservative surgery and reported that those with bilateral tumors tended to suffer from relapse within a shorter time (33.2 months for unilateral tumor and 23.0 months for bilateral tumors, *P* < 0.001). Results in this study indicated that bilateral tumors predicted a worse prognosis and decreased DFS, consistent with those results from previous literature.

Only 2 borderline ovarian tumor cases in this study had invasive implants. As a result, it is difficult to evaluate the impact of invasive implant on the prognosis of borderline ovarian tumor patients after fertility sparing surgery.

Chen RF et al. [16] reported that staging surgery only increased the FIGO stage but showed no influence on recurrence. Meanwhile, the pregnancy rate in patients diagnosed at an early stage and not undergoing staging surgery decreased from 81 to 54% (*P* = 0.08). In this study, up to 94% patients underwent cystectomy first before they underwent restaging surgery in our hospital upon the diagnosis of borderline ovarian tumors. Due to the limited data, the conclusion that staging surgery is not markedly associated with relapse or DFS among patients with borderline ovarian tumors should be interpreted with caution. Such a conclusion is similar to that of microinvasion, appendectomy and chemotherapy.

Obstetric outcomes among these groups are satisfying and similar at the last follow-up, and most of the patients had a good pregnancy rate (> 65%).

Above all, the advanced stage (≥stage II), serous type, presence of bilateral tumors and micropapillary pattern are significant factors leading to an increased risk of recurrence after conservative surgery. Borderline ovarian tumor patients with above characteristics should select fertility sparing surgery carefully and attempt to achieve pregnancy within a shorter time. In addition, it was reported in some studies that mucinous borderline ovarian tumors tend to progress into invasive ovarian cancer after conservative surgery [25, 26]. Therefore, the histology of mucinous type is not absolutely safe, and meticulous follow-up is needed for patients with mucinous borderline ovarian tumors and undergoing fertility sparing surgery.

Fertility sparing surgery can be performed in different ways. However, the oncological safety and fertility benefits of fertility sparing surgery remain unknown. The oncological and pregnancy outcomes of four fertility sparing surgery modalities (cystectomy, unilateral salpingo-oophorectomy, bilateral cystectomy, and unilateral salpingo-oophorectomy + contralateral cystectomy) were compared in this study. The results indicated that unilateral salpingo-oophorectomy was the optimal type of surgery, which was associated with the lowest recurrence rate and the longest recurrence interval. However, the difference in pregnancy rate among different fertility sparing surgery modalities was not statistically significant, which was in accordance with the results from previous studies. Compared with unilateral salpingo-oophorectomy, patients undergoing cystectomy had a higher pregnancy rate since more normal ovarian tissues were retained [27]. On the other hand, cystectomy may also increase the risk of recurrence and impact the DFS. The results of this study showed that the patients in the cystectomy group tended to have an obviously higher recurrence rate and a shorter recurrence interval (60% versus 24%, *p* = 0.123/36 months versus 55 months, *p* = 0.133), but the difference was not statistically significant. This study also indicated that both cystectomy and unilateral salpingo-oophorectomy groups had satisfying pregnancy rates (50% vs 69%, *P* = 1.000), but the cystectomy group showed no obvious fertility advantage. It was suggested by the univariate Cox regression analysis that the patients undergoing cystectomy had worse DFS than those receiving unilateral salpingo-oophorectomy, but such difference was not significant. The finding of this study is consistent other studies. For instance, Vasconcelos I et al. [28] found that unilateral salpingo-oophorectomy was notably favored over cystectomy, with an odds ratio for recurrence reduction of 2.20, 95% credibility interval of 0.793–2.841 and *p* < 0.0001. Notably, the surgical approach for bilateral borderline ovarian tumors remains uncertain due to the small sample size in this study. In this analysis, the recurrence rates of bilateral cystectomy and unilateral salpingo-oophorectomy + contralateral cystectomy were 50% and 67% (*P* = 1.000), respectively, inconsistent with the results from previous studies [16, 19, 23, 29–32]. This may be due to the small sample size of bilateral tumors in this study, since only 2 patients underwent bilateral cystectomy and 9 received unilateral salpingo-oophorectomy + contralateral cystectomy. Nonetheless, the pregnancy rate was still encouraging in both groups (100% versus 67%, P = 1.000). In addition, one study has reported that the pregnancy rate is notably higher in the bilateral cystectomy group than that in the unilateral salpingo-oophorectomy + contralateral cystectomy group (93% versus 53%) [33]. Therefore, bilateral cystectomy should be definitively favored in patients with bilateral borderline ovarian tumors due to its least aggressive nature. Subsequently, four fertility sparing surgery modalities were classified into two groups (cystectomy and adnexectomy) in this study, and the analysis showed that the surgical approach of adnexectomy was evidently favored over the cystectomy-including approach (HR = 3.3, 95% credibility interval = 1.338–8.140, *P* = 0.01). Additionally, the adnexectomy group had a lower recurrence rate (24% versus 63%, *P* = 0.012) and a longer recurrence interval (55 months versus 27 months, *P* = 0.007). Notably, both of the two groups showed good pregnancy rates (> 65%) among borderline ovarian tumor patients.

Additionally, a multivariate Cox regression model has been constructed in this study, which has incorporated the statistically significant factors, including FIGO stage, histology type, lesion lateral, presence of micropapillary lesion, and type of fertility preserving surgery, examined in the univariate analysis. Unfortunately, no factor was found to be significantly correlated with DFS, which may be caused by the insufficient sample size in this study. Consequently, studies including a large cohort size and a long-term follow-up period are needed to evaluate the correlation between above factors and the prognosis of borderline ovarian tumor patients.

Based on the results in this study, unilateral salpingo-oophorectomy should be recommended for patients with unilateral borderline ovarian tumors, whereas bilateral cystectomy, the least aggressive approach, should be recommended for patients with bilateral tumors.

## Conclusion

In this study, fertility sparing surgery was associated with an increased recurrence rate and decreased DFS compared with radical surgeries. Therefore, fertility sparing surgery should be recommended with caution. This retrospective analysis showed that an advanced stage (≥stage II), a serous type, and the presence of bilateral and micropapillary lesions were associated with a higher recurrence rate and a shorter recurrence interval after conservative surgery. The borderline ovarian tumor patients associated with the above characteristics should be more careful in choosing fertility sparing surgery and should attempt to achieve pregnancy as soon as possible. Additionally, the patients not yet ready to conceive can be referred to reproductive endocrinology and infertility clinics for other fertility options, including the freezing of embryos, oocytes and so on.

The results of this study suggest that adnexectomy (unilateral salpingo-oophorectomy) is preferable for patients with unilateral borderline ovarian tumors to achieve ideal oncological outcomes and a satisfactory pregnancy rate. In contrary, bilateral cystectomy, the least aggressive approach, should be preferably chosen for patients with bilateral borderline ovarian tumors, since no statistically significant difference in terms of recurrence rate, DFS or pregnancy outcome was observed between bilateral cystectomy and unilateral salpingo-oophorectomy + contralateral cystectomy groups.

Most of the previous retrospective studies employed short follow-up periods to investigate the outcomes of borderline ovarian tumor patients undergoing fertility sparing surgery. Consequently, more prospective studies with well-designed parameters, including a large cohort size and a long-term follow-up period, are needed to assess the safety and feasibility of fertility sparing surgery approaches.

## Abbreviations

DFS: Disease-free survival
FIGO: Federation of Gynecology and Obstetrics
HR: Hazard ratio
N/A: Not applicable
OS: Overall survival
PFS: Progression-free survival
WHO: World Health Organization
